# A qualitative examination of peer navigation for smoking cessation in people with HIV

**DOI:** 10.18332/tpc/213720

**Published:** 2025-12-16

**Authors:** Garrett S. Stang, Bianca Camacho, Megan Pinkston, Karen Tashima, Christopher W. Kahler, Patricia A. Cioe

**Affiliations:** 1 Center for Alcohol and Addiction Studies, Department of Behavioral and Social Sciences, Brown University School of Public Health, Providence, United States; 2 Department of Psychological Science, College of Liberal Arts, University of Texas Rio Grande Valley, Edinburg, United States; 3 Department of Psychiatry and Human Behavior, Warren Alpert Medical School of Brown University, Providence, United States; 4 Department of Medicine, Warren Alpert Medical School of Brown University, Providence, United States; 5 Division of Infectious Diseases, Warren Alpert Medical School of Brown University, Providence, United States; 6 College of Nursing, University of Rhode Island, Providence, United States

**Keywords:** smoking, cessation, peer navigation, HIV, qualitative research

## Abstract

**INTRODUCTION:**

People with HIV (PWH) are disproportionately affected by cigarette use, with a 40–60% prevalence rate. They achieve relatively low cessation rates following traditional interventions and often confront compounded challenges related to social factors. HIV care services have integrated Peer Navigators (PNs) into clinical care for many years, but not in the context of smoking cessation. The purpose of this study was to describe the experiences of PWH on a novel smoking cessation intervention that integrated PNs as part of a pilot randomized controlled trial.

**METHODS:**

This qualitative examination was conducted among PWH who smoke cigarettes and who participated in a randomized controlled trial between June 2020 and 2021 in Providence, Rhode Island, USA. A PN, defined as a PWH who smoked daily and successfully quit, was trained to provide cessation resources, encourage readiness to quit, and provide social support for quitting. Participants were randomized to either PN or usual care. Twenty-three participants assigned to a PN completed a semi-structured, in-depth qualitative interview. Interviews were audio-recorded, transcribed verbatim, and analyzed using thematic analysis.

**RESULTS:**

Analysis revealed that participants valued the interaction with the PN and described feeling increased social support for quitting. They expressed that the use of storytelling by the PN was linked to a sense of success, and that certain traits of the PN were perceived as salient. Interacting with a PN enforced a sense of accountability, and lead to feelings of enhanced self-efficacy.

**CONCLUSIONS:**

Integrating PNs to increase support for quitting seems to be highly acceptable among PWH who smoke. The findings underscore the significance of the lived experience of the peer navigator and the provision of social support.

## INTRODUCTION

Tobacco use remains the leading cause of preventable death and disease in the United States (US) despite the significant decline in commercial use over the past five decades^1^. People with HIV (PWH) are disproportionately affected by cigarette use, with an estimated 40–60% prevalence rate^2-4^, which is quadruple the 11.5% rate in the general population^1^. Advances in antiretroviral therapy (ART), along with engagement in HIV clinical care services, allow PWH to achieve an increased or near-normal lifespan^5^. However, the detrimental health effects of smoking lead to premature death from lung cancer and other smoking-related diseases. PWH who smoke have a higher incidence of comorbidities, including heart disease, cancer, and pulmonary disease, and lung cancer is now the leading cause of death among PWH, with tobacco use being the most significant risk factor for lung cancer incidence and mortality^2,6-10^. Indeed, prior research has determined that PWH lose more years of life due to smoking than to HIV-related morbidity^11,12^.

According to an evaluation of the beliefs and clinical practices of healthcare providers affiliated with the HIV Medical Association, clinicians agree that tobacco use is a critical issue in PWH^13^. Clinical tobacco treatment guidelines indicate that evidence-based behavioral and pharmacological interventions are still recommended for all people who smoke, including PWH^14^. However, previous smoking cessation intervention trials consistently report poor outcomes and lower success rates with traditional tobacco treatment, including poor adherence to therapy, despite PWH reporting great interest in tobacco treatment^13,15-22^. Considering their high levels of nicotine dependence and low quit rates, PWH who smoke may require additional support for quitting compared with the general population of smokers, as they may not only have more smokers in their social network but also may smoke to cope with social isolation related to living with HIV^23,24^.

Peer Navigators (PNs) have been utilized in the HIV care continuum for many years to support HIV prevention, testing, entry and retention in care, and adherence to ART^25-28^. Peers can use their personal experiences to establish meaningful connections with patients. They can then leverage these connections to inform, support, and retain patients in care^25^. Linking PWH who smoke with PNs (PWH who have quit smoking and remained smoke-free) could potentially increase motivation to quit, improve access to and utilization of available resources, and provide an example of mastery. This novel and sustainable approach can encourage smoking cessation and provide the additional social support necessary for successfully quitting and maintaining abstinence. The purpose of this qualitative study was to explore the experiences of PWH who received a PN-based intervention to provide social support for smoking cessation in a recent randomized clinical trial.

## METHODS

The parent study (n=64), was a pilot randomized controlled trial that examined the feasibility, acceptability, and preliminary efficacy of a peer navigation social support for smoking cessation intervention tailored for PWH who smoke cigarettes^29,30^. Participants were primarily recruited from an HIV care clinic in Providence, RI, and the local community. Eligible participants for the parent study were aged ≥18 years, diagnosed with HIV, smoked ≥5 cigarettes a day for more than a year, and tested positive for cotinine in their saliva at baseline. Participants were ineligible if they were already using smoking cessation pharmacotherapy, had an unstable medical or psychiatric condition (past 30-day medical or psychiatric hospitalization prior to enrollment), were experiencing psychotic symptoms, had past-month suicidal ideation or past-year suicide attempt, or were pregnant. Additionally, it is worth noting that participants did not have to endorse readiness to quit to be eligible.

Participants were enrolled June 2020–2021 and living in Providence, RI, USA. After their baseline visit, all participants were randomized upon completing a 30–45-minute smoking cessation counseling session with a study nurse. They then participated in follow-up interviews at 4, 12, and 24 weeks. The study methods and results of the RCT have been previously published^29,30^. All sessions were conducted via a HIPAA-compliant version of Zoom due to the COVID-19 pandemic. Briefly, the PN group received 12 weekly phone calls, lasting approximately 10–20 minutes each, from a PN following the initial session with the study nurse. These calls were manual-guided but not prescriptive – the PN was instructed to let the participant guide the conversation. One female PN who previously smoked daily for many years and had quit 1–2 years prior was hired for the study. She also had previous healthcare experience working as a nursing assistant. Abstinence was biochemically verified by an exhaled carbon monoxide (CO) level of ≤5 ppm at the time of hire. She received approximately 8 hours of didactic training (two 4-hour sessions conducted by PAC and MP), including role play. A manual was developed as a guide for the PN to deliver weekly phone sessions; including standard cessation counseling, local cessation resources, encourage readiness to quit, and social support for quitting. A more detailed overview of the PNs’ training, qualifications, and role, including details on participant phone call structure, is published elsewhere^29^.

The PN intervention concluded at the week 12 assessment. At week 12, 60-minute semi-structured, in-depth interviews were conducted via Zoom to examine the acceptability of the PN by active condition study participants. Twenty-seven out of thirty participants randomized to the PN condition attended the week 12 follow-up visit and were invited to complete the interview. Of those, 23 completed the interview. Descriptive statistics (means and measures of central tendency) were generated for select demographic, clinical, and smoking history variables collected via self-report in the parent study^30^. Data from the control group were not analyzed as those participants did not interact with the PN, and this report focuses solely on the experimental arms experience with the intervention.

The trained interviewers (research assistants on the project) queried participants’ perceptions of the PN, including perceived helpfulness. Interviews were audio-recorded, transcribed verbatim, and de-identified. Data management and analysis were conducted using NVivo version 11. Data were analyzed using an applied abductive thematic analysis approach (Supplementary file)^31^. A preliminary coding structure was created based on the semi-structured agenda and was refined as the coding process progressed to incorporate emerging themes. Codes were summarized, and five key themes were identified – illustrative quotes were chosen to represent each theme. The coding was conducted independently by the PI (PAC) and a medical student research assistant (BC), both of whom were not involved in the delivery of the intervention or assessing outcomes to ensure objectivity in the qualitative analysis. Coding discrepancies in codes were resolved through consensus.

Participants were asked: ‘Tell me what you liked and/or disliked about your peer navigator’; ‘Can you give one example of helpfulness or one thing they said that was helpful? One great piece of advice?’; ‘Can you tell me, in more detail, one thing the PN addressed with you?’; and ‘Would you have preferred a male or female PN? Why or why not? How might that have made a difference? How important was it that the PN be the same gender as you?’.

### Ethical considerations

The Brown University Human Research Protection Program/Institutional Review Board (IRB) and Brown University Health System IRB (previously Lifespan) approved this study (STUDY00000556) on 6 August 2019. All participants provided written informed consent and were compensated for their participation.

## RESULTS

Twenty-three participants (76.7%) in the PN arm who attended the week 12 follow-up completed an interview. The four participants who did not participate did not provide a reason for declining. Table 1 summarizes the sample demographic and smoking characteristics. On average, participants were aged 57 years (SD=7.4), White (52.2%), and female (60.9%). Participants actively engaged with the PN, with a mean number of 9.5 (SD=2.4) weekly calls completed (out of 12 possible calls). One quarter (n=7; 23.3%) of participants completed all 12 weekly calls with the PN, while 50% completed 10 calls. The initial efficacy of the PN intervention showed promising results for increased social support for quitting. By week 24, 16 participants (53.3%) in the PN group reported making a quit attempt, and five self-reported (CO-biochemically verified) as having successfully quit. Interestingly, the quit rates did not differ between the study conditions. However, as anticipated, participants in the active condition experienced a significant increase in positive social support from baseline to week 12. This included enhanced self-efficacy for quitting, which also improved over time, although not significantly when compared to the control group.

**Table 1:** Characteristics and baseline smoking information of participants who completed a qualitative interview from a peer navigation intervention for smoking cessation RCT (N=23)

*Characteristics*	*n (%)*
**Sex at birth**	
Female	14 (60.9)
Male	9 (39.1)
**Race**	
White	12 (52.2)
Black	7 (30.4)
Other	4 (17.3)
**Ethnicity**	
Hispanic	6 (26.1)
Non-Hispanic	17 (73.9)
	***Mean (SD)***
Age (years)	57.09 (7.35)
Education (years)^a^	11.91 (1.98)
Years living with HIV	22.87 (10.25)
Cigarettes per day	16.41 (9.87)
Years smoking cigarettes	36.09 (11.87)
Cigarette dependence (FTCD)^b^	6.04 (2.01)
Confidence to quit (scale 1–10)	7.04 (2.38)
Completed PN Calls^c^	9.48 (2.37)

a Education presented as total number of years completed.

b FTCD: Fagerström test for cigarette dependence (scores range 1–10, higher scores indicate higher dependence on cigarettes/nicotine).

c PN: peer navigator.

The five themes that were generated from the interview data describe several key aspects of the PN and participant interaction, including: 1) social support through peer navigation; 2) storytelling for success; 3) perceived salient traits of PN; 4) accountability; and 5) enhanced self-efficacy.

### Social support through peer navigation

The PN provided valuable support for individuals looking to achieve or maintain abstinence. This support was due to a reduced sense of isolation, having a partner in their quitting journey, and a positive role model for cessation:

*‘She just gave me that “you’re not alone” kind of feeling.’* (Male, 58 years)

*‘She’s very consistent. Reliable. She was very upbeat. Positive. And she was caring and genuinely concerned. She provided a lot of helpful resources. And you know, spoke to me as if we were best friends. To me that’s the kind of support anybody would want.’* (Female, 42 years)

Some participants expressed anxiety about ‘losing’ the PN after the study was completed:

*‘I’m mad that the next week I talk to her is the last week.’* (Male, 49 years)

*‘I feel bad I’m going to be losing her soon.’* (Female, 63 years)

This reflects the strong bond formed between the participants and their PN and underscores the need for the PN to help participants establish a similar support system within their own networks.

### Storytelling for success

The PN’s ability to recall personal anecdotes from her journey from smoking to abstinence strengthened the bond between the PN and the participants. These tips gave participants a new perspective on quitting smoking that they may only have considered with the guidance of a former smoker:

*‘Explaining to me how to use study products. Given me other ideas how to quit. Ya know, coaching me.’* (Male, 63 years)

*‘We opted for sugar-free hard candies and placed strategically around my apartment so that I always had easy access to something.’* (Male, 58 years)

*‘She teaches me new techniques.’* (Male, 37 years)

*‘She told me to wash my wall down and get rid of the ashtrays. I thought that was really cute, but it actually helped!’* (Female, 55 years)

*‘I think maybe [name] had her mind set on one option and that was the Lozenger (sic). She would kinda, ya know, push for the Lozenger and I thought at one point it was a little too salesmen-y … but that was minor, really.’* (Female, 60 years)

These testaments also underscore the importance of the PN offering multiple smoking cessation options for participants to try rather than advocating for a single method based solely on their personal experience.

### Perceived salient traits of peer navigator

Salient traits of the PN shaped participants’ overall perceptions and experiences of the weekly phone calls. Participants consistently highlighted attributes such as:

Authenticity

*‘A peer navigator is someone who is going through the same thing. They’re not just “talking theory” to you.’* (Male, 66 years)

*‘[About if the Peer Navigator should also be HIV positive] Um, it depends on who they’re trying to help. I mean of course it would be beneficial if the person that they was trying to help also had HIV. And maybe someone that wasn’t would feel that extra “not only has this person stopped smoking but he’s also dealing with these other things. If he or she can do that, then I can rise above this smoking too”. It depends on the person.’* (Male, 66 years)

Candidness

*‘She tells it like it is. Whether you like it or not. I like that.’* (Male, 57 years)

*‘[About having a peer navigator] It gives you the opportunity to bounce ideas off that person.’* (Male, 66 years)

Reliability

*‘She never missed calling me.’* (Female, 67 years)

When asked about their preference for sex- or gender-identity-matching their PN, participants did not perceive this as a salient trait. Most said it did not make a difference and often underscored that merely having access to a PN was the support they needed:

*‘It doesn’t matter, it’s all about the support system.’* (Male, 56 years)

*‘It didn’t really matter. I, I, I just needed that push that extra [support] ... and I got it from her.’* (Male, 49 years)

*‘No it don’t matter. It depends on the [PN] and how they got confidence and how they talk to you.’* (Male, 54 years)

### Accountability

Participants reported that the PN was beneficial in reminding them about important events such as appointments, medication management, and quit dates. The majority of participants felt that a regular call from the PN brought smoking cessation to the forefront of their minds:

*‘She was helpful because she had me remembering my appointments.’* (Female, 52 years)

*‘Talking to [PN] every week has made me more aware.’* (Female, 42 years)

While most participants stressed the effect of accountability, others felt pressured by the phone calls, highlighting the importance of individualizing counseling:

*‘I think once a week creates pressure and obligation and if you don’t have anything good to say then you’re gonna feel bad saying it. If you don’t have to say “I’ve been a failure” every week as opposed to once a month you’re better off (laughs).’* (Male, 65 years)

### Enhanced self-efficacy

Interactions with the PN increased participants’ self-confidence and reinforced optimism. Observing the vicarious experiences of others and receiving verbal persuasion enhanced participants’ self-efficacy to quit:

*‘Knowing what she herself was going through was very helpful.’* (Male, 48 years)

*‘She gives me confidence.’* (Male, 54 years)

*‘I don’t know if it’s really advice, but the fact that she found the positive even when I wasn’t able to see it.’* (Female, 57 years)

*‘When she talks to you, she believes in you, you know what I’m saying?’* (Male, 54 years)

*‘She’s very informative. Any sort of thing that made me uneasy she was great at finding the right information for me.’* (Male, 58 years)

## DISCUSSION

The findings from this study support that participants found substantial value in receiving support for smoking cessation from a PN. In particular, they spoke to how the PN provided social support for quitting and increased their self-efficacy. According to social cognitive theory, these two outcomes may facilitate smoking cessation since both can influence the ability to take on a new challenge (such as smoking cessation) and persevere in time of difficulty (such as cravings)^32^. The PN, as a person living with HIV themselves and who formerly smoked, also may have provided an example of mastery – someone who has faced similar challenges and has maintained smoking abstinence, which some participants explicitly noted. Further, the theory of triadic influence also emphasizes that streams of influence, such as those found in social networks, influence the individual’s attitudes and behavioral choices^33^. This is particularly relevant for PWH, as they often report that their social networks, including friends and family, include many smokers, which can hinder their self-efficacy to quit^34^.

A key point that is especially relevant and was emphasized in the interviews was described by one participant as ‘talking theory’. This highlights the belief that the PN was a more credible source of information due to their lived experience as a former person who smoked. While healthcare providers often recommend smoking cessation to their patients with HIV, many providers lack the personal experience that the PN brings, which can make their advice feel less impactful. They may struggle to connect as deeply with patients who smoke. In contrast, participants held the PN in high regard for their honesty, authenticity, and credibility as someone who has quit smoking. Their sincerity fostered a deeper connection with the participants that healthcare staff might be unable to achieve. The bond that developed over time between the participant and the PN fostered a ‘feeling’ of community the participants appreciated, highlighting the importance of encouragement and rallying support that PWH may need to start the process of reducing cigarette smoking, staying abstinent, or combatting cravings.

The importance of social support and self-efficacy in promoting medication adherence (e.g. ART) among PWH is well-established^35^. The PN in our trial provided salient social support and education related to cessation efforts and access to treatment resources (e.g. quitline, and nicotine replacement therapy [NRT]), emphasizing the positive influence of perceived support through shared lived experiences surrounding access and use of available treatment options. Our findings that real-time social support may promote continuous abstinence align with a wealth of previous work^35^, and are consistent with recent findings of Shuter et al.^36^.

We trained one peer navigator who identified as female and had previous experience working in healthcare as a nursing assistant; thus, she may have had more well-developed communication skills. To our knowledge, this is the first study to qualitatively examine the acceptability of cross-training HIV clinic peer navigators to provide smoking cessation counseling and social support for quitting.

### Limitations

The study has some limitations. The sample size was small, and participants were recruited from a single city, working with one peer navigator. A notable limitation is that participants were required to utilize Zoom, which is contingent upon access to an electronic device, internet access, and a private space to participate in the interview. Therefore, aside from limiting representativeness in the sample, there is potential for mode administration bias. As a result, the findings may not be generalizable to all settings and could vary depending on the qualities and characteristics of different peer navigators. Findings also may not generalize to all PWH who smoke. While participants in this study reported that gender identity matching was not an important salient trait contributing to the relationship with their PN, not all PWH may feel the same way. This will require larger samples with multiple trained PNs to thoroughly assess whether gender identity matching and pairing is a key factor in achieving successful outcomes.

## CONCLUSIONS

The findings of this study are promising as this qualitative data demonstrates that cross-training and utilizing PNs in smoking cessation programs for PWH who smoke appears to be highly acceptable by clinic patients who express interest in smoking cessation. The data suggest that the PN provided social support and education that was salient. Consistent with the underlying theory, the guidance, advice, and support from the PN were perceived as authentic and impactful, increasing self-efficacy for quitting. Training peer navigators in HIV care clinics to offer social support for smoking cessation could be a cost-effective way to leverage an underutilized clinical resource and enhance smoking cessation outcomes for this population. However, a future clinical trial would be needed to test whether social support and accountability do, in fact, act as mechanisms of action for PN and whether sex- or gender-identity-matching does not, in fact, affect responses to PN.

## Supplementary Material



## Data Availability

The data supporting this research are available from the authors on reasonable request.
