# Supplementary material for: A qualitative examination of peer navigation for smoking cessation in people with HIV
Source: Tob Prev Cessat. 2025 Dec 16;11:10.18332/tpc/213720. doi: 10.18332/tpc/213720 (PMC12707165; doi:10.18332/tpc/213720)
Supplement: Supplementary file 1 [file TPC-11-58-s1.pdf]

Consolidated criteria for reporting qualitative studies (COREQ): 32-item checklist

No. Item	Guide questions/description	Reported on Page #
**Domain 1: Research team and reflexivity**		
Personal Characteristics		
1. Inter viewer/facilitator	Which author/s conducted the interview or focus group?	**p.3**
2. Credentials	What were the researcher's credentials? E.g. PhD, MD	**p.3; Masters**
3. Occupation	What was their occupation at the time of the study?	**p.3; Research Assistant**
4. Gender	Was the researcher male or female?	**Female**
5. Experience and training	What experience or training did the researcher have?	**p.3; Masters public health/behavioral and social health science level training**
Relationship with participants		
6. Relationship established	Was a relationship established prior to study commencement?	**p.3; Yes, indirectly as the study RA**
7. Participant knowledge of the interviewer	What did the participants know about the researcher? e.g. personal goals, reasons for doing the research	**Minimal; focus was on PN, not interviewer**
8. Interviewer characteristics	What characteristics were reported about the inter viewer/facilitator? e.g. Bias, assumptions, reasons and interests in the research topic	**p.3; independence from intervention team and thematic analysis team**

**Domain 2: study design**		
Theoretical framework		
9. Methodological orientation and Theory	What methodological orientation was stated to underpin the study? e.g. grounded theory, discourse analysis, ethnography, phenomenology, content analysis	**p.3; applied thematic analysis**
Participant selection		
10. Sampling	How were participants selected? e.g. purposive, convenience, consecutive, snowball	**p.3; parent RCT**
11. Method of approach	How were participants approached? e.g. face-to-face, telephone, mail, email	**Described in parent RCT**
12. Sample size	How many participants were in the study?	**p.4; 23**
13. Non-participation	How many people refused to participate or dropped out? Reasons?	**p.4; Four with no reasons reported**
Setting		
14. Setting of data collection	Where was the data collected? e.g. home, clinic, workplace	**p.3; Zoom**
15. Presence of non-participants	Was anyone else present besides the participants and researchers?	**p.3; No**
16. Description of sample	What are the important characteristics of the sample? e.g. demographic data, date	**p.4; 13 (Table 1)**
Data collection		
17. Interview guide	Were questions, prompts, guides provided by the authors? Was it pilot tested?	**p.3**
18. Repeat interviews	Were repeat inter views carried out? If yes, how many?	**N/A**
19. Audio/visual recording	Did the research use audio or visual recording to collect the data?	**p.3; audio**
20. Field notes	Were field notes made during and/or after the inter view or focus group?	**Minimal**
21. Duration	What was the duration of the inter views or focus group?	**p.3; 60-min**

22. Data saturation	Was data saturation discussed?	**p.No since any participant in the parent study was eligible to complete**
23. Transcripts returned	Were transcripts returned to participants for comment and/or correction?	**No**
**Domain 3: analysis and findings**		
Data analysis		
24. Number of data coders	How many data coders coded the data?	**p.3; two**
25. Description of the coding tree	Did authors provide a description of the coding tree?	**No**
26. Derivation of themes	Were themes identified in advance or derived from the data?	**p.3; yes abductive approach**
27. Software	What software, if applicable, was used to manage the data?	**p.3; Nvivo**
28. Participant checking	Did participants provide feedback on the findings?	**No**
Reporting		
29. Quotations presented	Were participant quotations presented to illustrate the themes/findings? Was each quotation identified? e.g. participant number	**p.4-6; yes**
30. Data and findings consistent	Was there consistency between the data presented and the findings?	**Yes**
31. Clarity of major themes	Were major themes clearly presented in the findings?	**Yes**
32. Clarity of minor themes	Is there a description of diverse cases or discussion of minor themes?	**Yes**

© 2025 Stang G.S. et al.
